# Association of loneliness with incident cardiovascular diseases in middle-aged and older Chinese adults

**DOI:** 10.1371/journal.pone.0343021

**Published:** 2026-02-23

**Authors:** Bo Xie, Yehua Fang, Fang Lin, Ming Yang, Yanchao Liang, Bin Liu

**Affiliations:** 1 Department of Scientific Research, Zhuzhou Central Hospital, Zhuzhou Hospital Affiliated to Xiangya School of Medicine, Central South University, Zhuzhou, Hunan, China; 2 Department of Clinical Psychology, Zhuzhou Central Hospital, Zhuzhou Hospital Affiliated to Xiangya School of Medicine, Central South University, Zhuzhou, Hunan, China; 3 Department of Obstetrics and Gynecology, Liuyang People’s Hospital, Nanhua University, Liuyang, Hunan, China; 4 Department of Nephrology, Zhuzhou Central Hospital, Zhuzhou Hospital Affiliated to Xiangya School of Medicine, Central South University, Zhuzhou, Hunan, China; 5 Department of Respiratory and Critical Care Medicine, Zhuzhou Central Hospital, Zhuzhou Hospital Affiliated to Xiangya School of Medicine, Central South University, Zhuzhou, Hunan, China; University of Georgia, UNITED STATES OF AMERICA

## Abstract

Loneliness, a significant social determinant of health, has been increasingly recognized for its potential to influence cardiovascular disease (CVD) risk. This study aimed to delineate the relationship between loneliness and the incidence of CVD, specifically heart disease and stroke, among Chinese adults aged 45 and older. Utilizing data from the China Health and Retirement Longitudinal Study (CHARLS), loneliness was assessed using a validated single-item measure. Incident CVD events, including heart disease and stroke, were ascertained through standardized interviews. We employed Cox proportional hazards models to calculate hazard ratios (HRs), adjusting for a comprehensive set of covariates. A longitudinal analysis of 8,046 participants initially free of CVD was conducted. During a 7-year follow-up period, 1,033 participants experienced incident CVD events. Loneliness was found to be an independent risk factor for CVD, with individuals reporting loneliness exhibiting a 42.9% higher risk of developing CVD (adjusted HR: 1.429; 95% CI, 1.251–1.632). Specifically, the risk of heart disease was elevated by 44.7% (adjusted HR: 1.447; 95% CI, 1.229–1.703), and the risk of stroke was increased by 27.9% (adjusted HR: 1.279; 95% CI, 1.035–1.580). This association was particularly pronounced among those without a history of hypertension, where the risk of CVD was elevated by 58.2% (adjusted HR: 1.582; 95% CI, 1.317–1.900). Our findings indicate that loneliness is significantly associated with an increased risk of CVD, specifically heart disease and stroke, among Chinese adults aged 45 and older. These results highlight the need to consider psychosocial factors in CVD prevention strategies.

## Introduction

Cardiovascular disease (CVD) stands as a leading cause of death worldwide [1,2], encompassing a wide array of conditions that impact the heart and blood vessels [3]. This poses a significant public health challenge, with the expected rise in CVD prevalence due to the global aging population [4]. This trend has far-reaching consequences for personal health and imposes a considerable burden on healthcare systems [5,6]. Hence, gaining a deeper insight into the pathogenesis of CVD and identifying modifiable risk factors are pivotal for effective prevention and control measures.

Loneliness, understood as the subjective sensation of social isolation, has been associated with various negative health impacts [7,8]. Research suggests that loneliness not only correlates with mental health challenges, including depression and anxiety, but also with physical health issues, notably CVD [9,10]. The constant activation of physiological stress responses triggered by loneliness can affect inflammation and immune responses, thus heightening the risk of CVD and related fatalities [11,12]. Additionally, loneliness has been linked to poor weight management, elevated tobacco usage, reduced physical activity, and compromised sleep quality [13–15], all of which indirectly elevate the CVD risk. A study conducted in the United States revealed that social isolation and loneliness, when considered independently, are linked to a moderately elevated CVD risk in postmenopausal women [16]. Notably, women experiencing both conditions simultaneously had a higher CVD risk compared to those dealing with either factor alone. Another investigation showed that diabetic patients who feel lonely are at a greater risk of cardiovascular disease, with this risk increasing in proportion to the degree of risk factor control [9]. While Zheng and colleagues offered significant perspectives on the combined influence of vulnerability and loneliness on CVD, their study did not explore in-depth the effect of loneliness on CVD, independent of other vulnerability factors [8].

In China, the relevance of loneliness in middle-aged and older adults is heightened by unique cultural and societal factors. Rapid urbanization and the ‘empty nest’ phenomenon have led to increasing an number of older adults living apart from their children, potentially exacerbating feelings of loneliness and social isolation. Additionally, the traditional cultural emphasis on family cohesion and interdependence may create specific challenges for individuals experiencing loneliness, as the stigma associated with expressing such feelings might prevent them from seeking social support. Moreover, compared to Western populations, there is a relative lack of research specifically focusing on the health impacts of loneliness in Chinese middle-aged and older adults, particularly in the context of cardiovascular disease. This gap in evidence limits our understanding of how cultural and social determinants shape the loneliness-CVD relationship in this specific population, and highlights the need for more targeted studies to inform culturally appropriate prevention strategies.

The goal of this study is to investigate the prospective association between loneliness and the incidence of cardiovascular disease, and to evaluate the potential risk of CVD associated with loneliness in middle-aged and older Chinese adults using Cox proportional hazards models.

## Methods

### Study population

The study utilized data from the China Health and Retirement Longitudinal Study (CHARLS), a national, multicenter, longitudinal survey aimed at collecting comprehensive information on the health, economic, and social conditions of middle-aged and older adults in China [17,18]. CHARLS was initiated in 2011–2012, employing a multistage stratified random sampling method to select 150 counties/districts and 450 villages/communities across 28 provinces in China. Over 17,700 adults aged 45 years and older were recruited as initial participants. Follow-ups were conducted every two years, with four rounds completed by the end of 2018. Participants provided detailed personal information through face-to-face interviews, including sociodemographic characteristics, lifestyle habits, health status, and mental health.

**Fig 1:**
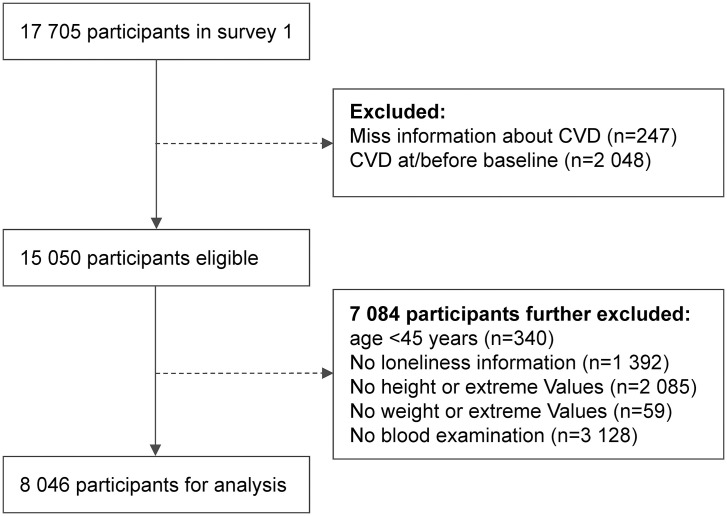
Flow chat of participants in the cohort study.

Participants for this study were selected based on the following criteria: age 45 years or older; no history of cardiovascular disease (CVD) at baseline; complete data on loneliness; and complete follow-up information on CVD. In terms of BMI, participants with extreme values (BMI < 15 kg/m² or > 40 kg/m²) were excluded to ensure the representativeness and reliability of the study population, as these extremes may indicate other severe health issues rather than general variations in body weight. After screening, a total of 8,046 participants were included in the final analysis. The selection process of the study population is illustrated in **Fig 1**.

### Ethical approval

The CHARLS study was approved by the Ethics Review Committee of Peking University (IRB00001052-11014, IRB00001052-11015). All participants provided written informed consent before enrollment. The present secondary analysis used de-identified, publicly available CHARLS data and was exempt from additional ethical review according to institutional guidelines.

### Assessment of exposure

To measure loneliness, a simplified approach was adopted using one question from the Center for Epidemiologic Studies Depression Scale (CESD): “How often did you feel lonely in the past week?” Participants rated their feelings on a 4-point scale ranging from “never” to “always”. Despite the potential underestimation of true loneliness prevalence due to single-item self-reporting, this method was chosen for its simplicity and widespread applicability. The single-item measure has been widely used in large-scale epidemiological studies, such as the CES-D scale from which this question originated. It provides a concise yet effective way to assess loneliness in a large population, and its reliability and validity have been confirmed in previous studies [19,20].

For analysis, loneliness was dichotomized into two categories: no loneliness – rarely or never feeling lonely; and loneliness – sometimes, occasionally, or frequently feeling lonely.

### Assessment of outcomes

The primary outcome was incident CVD events, including heart disease and stroke. Incident CVD events were determined using standardized questions: “Has a doctor ever told you that you have heart disease (e.g., myocardial infarction, coronary artery disease, angina, congestive heart failure, or other heart problems) or stroke?” Participants who reported heart disease or stroke during follow-up were considered to have experienced an incident CVD event. The date of the incident CVD event was recorded as the midpoint between the last visit date and the visit date when the event was reported. All participants contributed to the first recorded CVD outcome, excluding subsequent CVD deaths after non-fatal CVD events [21,22].

### Assessment of covariates

We considered a comprehensive set of covariates to control for various factors potentially influencing CVD risk. At baseline, trained interviewers collected detailed information on sociodemographic characteristics and health-related factors through structured questionnaires. Variables included age (recorded in years), sex (male and female), residence (rural vs. non-rural household registration), marital status [married vs. other (unmarried, separated, divorced, and widowed)], education level (illiterate, primary school, middle school or above), smoking status (never smoked, former smoker, current smoker), and alcohol consumption (yes or no).

Additionally, comorbidities including hypertension, diabetes, and hyperlipidemia were collected. Hypertension was defined as systolic blood pressure ≥140 mmHg or diastolic blood pressure ≥90 mmHg, or a self-reported history of hypertension. Diabetes was defined based on fasting glucose ≥7.0 mmol/L or a self-reported diagnosis of diabetes. Body mass index (BMI) was calculated as weight (kg) divided by height (m²) and categorized as underweight (BMI < 18.5 kg/m²), normal weight (18.5 kg/m² ≤ BMI < 24 kg/m²), and overweight or obese (BMI ≥ 24 kg/m²).

Metabolic markers such as total cholesterol, triglycerides, low-density lipoprotein cholesterol, high-density lipoprotein cholesterol, glucose, glycosylated hemoglobin HbA1c, and C-reactive protein (CRP) were obtained for a subset of 8,696 participants through laboratory tests, providing important biomarkers for assessing individual cardiovascular health risks.

### Statistical analysis

Data were described as means and standard deviations (SDs) for normally distributed continuous variables, medians and interquartile ranges for non-normally distributed continuous variables, and frequencies and percentages for categorical variables. Baseline characteristics were summarized by loneliness status and compared using χ² tests, analysis of variance, or Mann-Whitney U tests. For missing data, we assumed data were missing at random and used multiple imputation by chained equations (MICE).

Follow-up time was calculated from the baseline survey date in 2011–2012 to the date of diagnosis of CVD, stroke, or heart events, or the end of follow-up in 2018, whichever occurred first. Incidence rates of CVD, stroke, and heart events per 1,000 person-years were computed according to loneliness status.

Kaplan-Meier curves and log-rank tests were used to compare cumulative event risks between groups. Cox proportional hazards models were used to estimate hazard ratios (HRs) and 95% confidence intervals (CIs) for CVD, stroke, and heart events. Three levels of covariate adjustment were performed in the Cox models: Model 1 adjusted for age and sex; Model 2 added adjustment for residence, marital status, education level, smoking status, and alcohol consumption; and Model 3 further adjusted for systolic blood pressure, BMI, diabetes, hypertension, and hyperlipidemia.

Subgroup analyses were conducted to examine whether the association between loneliness and CVD, stroke, and heart events was modified by demographic and clinical characteristics, including age, sex, residence, marital status, education level, smoking status, alcohol consumption, diabetes, hypertension, hyperlipidemia, and BMI. Interaction terms and likelihood ratio tests were used to assess interaction p-values. Sensitivity analyses using inverse probability of treatment weighting (IPTW) were conducted to validate the robustness of the results.

All statistical analyses were performed using SPSS version 26.0 and R version 4.2.2. Two-sided p-values less than 0.05 were considered statistically significant.

## Results

### Baseline characteristics

A total of 8,046 adults were included in the analyses. The median (IQR) age at baseline was 58.00 (52.00, 65.00) years; 3,834 (47.7%) of the participants were men and 4,212 (52.3%) were women. **Table 1** shows the characteristics of the participants. At baseline, 2,227 participants (27.7%) reported feelings of loneliness. Univariate analysis revealed that compared with those without loneliness, participants with loneliness were more likely to be older (median [IQR] age, 59.00 [53.00, 66.00] vs 58.00 [51.00, 64.00] years; p < 0.001), be female (59.0% vs 49.8%; p < 0.001), live in a rural setting (88.4% vs 82.1%; p < 0.001), be unmarried (23.1% vs 7.2%; p < 0.001), have lower education levels (illiterate, 34.3% vs 26.6%; primary school, 42.8% vs 40.7%; p < 0.001), be never smokers (64.0% vs 58.9%; p < 0.001), drink less alcohol (68.8% vs 64.7%; p = 0.001), and have a lower body mass index (BMI) (median [IQR], 22.63 [20.44, 25.17] kg/m² vs 23.18 [20.93, 25.78] kg/m²; p < 0.001).

**Table 1:** Baseline characteristics of participants with and without loneliness.

Characteristics	Overall	No loneliness	Loneliness	*p* value
**N (%)**	8046	5819	2227	
**Age, years [IQR]**	58.00 [52.00, 65.00]	58.00 [51.00, 64.00]	59.00 [53.00, 66.00]	<0.001
**Gender, n(%)**				<0.001
Male	3834 (47.7)	2922 (50.2)	912 (41.0)	
Female	4212 (52.3)	2897 (49.8)	1315 (59.0)	
**Residence, n(%)**				<0.001
Rural	6747 (83.9)	4779 (82.1)	1968 (88.4)	
Other	1299 (16.1)	1040 (17.9)	259 (11.6)	
**Marital status, n(%)**				<0.001
Married	7111 (88.4)	5399 (92.8)	1712 (76.9)	
Other	935 (11.6)	420 (7.2)	515 (23.1)	
**Smoking status, n(%)**				<0.001
Never	4853 (60.3)	3427 (58.9)	1426 (64.0)	
Former	666 (8.3)	502 (8.6)	164 (7.4)	
Current	2527 (31.4)	1890 (32.5)	637 (28.6)	
**Drinking status, n(%)**				0.001
No	5299 (65.9)	3766 (64.7)	1533 (68.8)	
Yes	2747 (34.1)	2053 (35.3)	694 (31.2)	
**Education level, n(%)**				<0.001
Illiterate	2312 (28.7)	1548 (26.6)	764 (34.3)	
Primary school	3321 (41.3)	2368 (40.7)	953 (42.8)	
Second/high school and above	2413 (30.0)	1903 (32.7)	510 (22.9)	
**BMI, kg/m2 [IQR]**	23.04 [20.79, 25.62]	23.18 [20.93, 25.78]	22.63 [20.44, 25.17]	<0.001
**BMI category, n (%)**				<0.001
Underweight	540 (6.7)	345 (5.9)	195 (8.8)	
Normal weight	4331 (53.8)	3085 (53.0)	1246 (55.9)	
Overweight or obesity	3175 (39.5)	2389 (41.1)	786 (35.3)	
**DBP, mmHg [IQR]**	74.67 [67.00, 83.00]	75.00 [67.33, 83.33]	74.33 [67.00, 82.67]	0.277
**SBP, mmHg [IQR]**	127.00 [114.33, 142.00]	127.00 [114.67, 141.67]	127.00 [114.00, 143.33]	0.819
**Hypertension, n(%)**				0.228
No	4957 (61.6)	3609 (62.0)	1348 (60.5)	
Yes	3089 (38.4)	2210 (38.0)	879 (39.5)	
**Diabetes, n(%)**				0.934
No	7462 (92.7)	5398 (92.8)	2064 (92.7)	
Yes	584 (7.3)	421 (7.2)	163 (7.3)	
**Dyslipidemia, n(%)**				0.910
No	6093 (75.7)	4409 (75.8)	1684 (75.6)	
Yes	1953 (24.3)	1410 (24.2)	543 (24.4)	
**Metabolic biomarkers**				
Total cholesterol, mg/dL [IQR]	1.15 [0.82, 1.67]	1.14 [0.82, 1.68]	1.15 [0.83, 1.65]	0.891
Triglycerides, mg/dL [IQR]	190.59 [167.40, 215.63]	190.21 [167.40, 214.56]	191.37 [167.01, 218.24]	0.086
LDL cholesterol, mg/dL [IQR]	114.05 [93.17, 137.24]	114.05 [93.17, 136.47]	114.43 [93.17, 139.18]	0.197
HDL cholesterol, mg/dL [IQR]	49.48 [40.59, 59.92]	49.10 [40.21, 59.54]	51.03 [41.75, 61.47]	<0.001
Glucose, mg/dL [IQR]	102.42 [94.32, 113.22]	102.42 [94.50, 113.22]	102.42 [94.23, 112.86]	0.492
HBA1C, % [IQR]	5.10 [4.90, 5.40]	5.10 [4.90, 5.40]	5.10 [4.90, 5.40]	0.697
CRP, mg/L [IQR]	1.02 [0.54, 2.13]	1.03 [0.55, 2.13]	1.00 [0.54, 2.12]	0.487

Abbreviation: N, number; IQR, interquartile range; BMI, body mass index; DBP, diastolic blood pressure; SBP, systolic blood pressure; LDL, low-density lipoprotein; HDL, high-density lipoprotein; HbA1c, glycated hemoglobin; CRP, C-reactive protein.

Participants with loneliness were also more likely to be underweight (8.8% vs 5.9%; p < 0.001) and less likely to be overweight or obese (35.3% vs 41.1%; p < 0.001) compared to those without loneliness. There were no significant differences in diastolic blood pressure (DBP) (74.33 [67.00, 82.67] mmHg vs 75.00 [67.33, 83.33] mmHg; p = 0.277), systolic blood pressure (SBP) (127.00 [114.00, 143.33] mmHg vs 127.00 [114.67, 141.67] mmHg; p = 0.819), history of hypertension (39.5% vs 38.0%; p = 0.228), or prevalence of diabetes (92.7% vs 92.8%; p = 0.934) between the two groups. However, participants with loneliness had higher levels of high-density lipoprotein (HDL) cholesterol (median [IQR], 51.03 [41.75, 61.47] mg/dL vs 49.10 [40.21, 59.54] mg/dL; p < 0.001) compared to those without loneliness.

### Association of loneliness with incident cardiovascular disease

During the follow-up period from 2011 to 2018, a total of 1,033 participants experienced incident CVD, including 679 cases of heart disease and 419 cases of stroke. The sum of heart disease and stroke cases exceeds the total number of CVD cases because some participants experienced both conditions. The incidence rates of CVD were 22.58 per 1,000 person-years among participants with loneliness and 16.72 per 1,000 person-years among those without loneliness. After adjusting for potential confounders (Model 3), the presence of loneliness was independently associated with a 42.9% increased risk of incident CVD (adjusted HR, 1.429; 95% CI, 1.251–1.632), a 44.7% increased risk of heart disease (adjusted HR, 1.447; 95% CI, 1.229–1.703), and a 27.9% increased risk of stroke (adjusted HR, 1.279; 95% CI, 1.035–1.580). The results are summarized in **Table 2**.

**Table 2:** Incidence of cardiovascular diseases according to loneliness.

Outcome	Cases, No.	Incidence Rate, per 1000 Person-Years	HR (95% CI)		
			Model 1^a^	Model 2^b^	Model 3^c^
Cardiovascular disease					
No loneliness	681	16.72	1 [Reference]	1 [Reference]	1 [Reference]
Loneliness	352	22.58	1.321 (1.160, 1.503)	1.394 (1.221, 1.591)	1.429 (1.251, 1.632)
Heart disease					
No loneliness	448	11.00	1 [Reference]	1 [Reference]	1 [Reference]
Loneliness	231	14.82	1.303 (1.111, 1.529)	1.414 (1.201, 1.664)	1.447 (1.229, 1.703)
Stroke					
No loneliness	282	6.92	1 [Reference]	1 [Reference]	1 [Reference]
Loneliness	137	8.79	1.024 (1.014, 1.034)	1.245 (1.008, 1.538)	1.279 (1.035, 1.580)

Abbreviation: HR, hazard ratio.

^a^ Model 1 was adjusted for age and sex.

^b^ Model 2 was adjusted for age, sex, residence, marital status, educational level, smoking status, and drinking status.

^c^ Model 3 was adjusted as model 2 plus systolic blood pressure and body mass index, history of diabetes, hypertension, and dyslipidemia.

### Subgroup analyses

**Fig 2:**
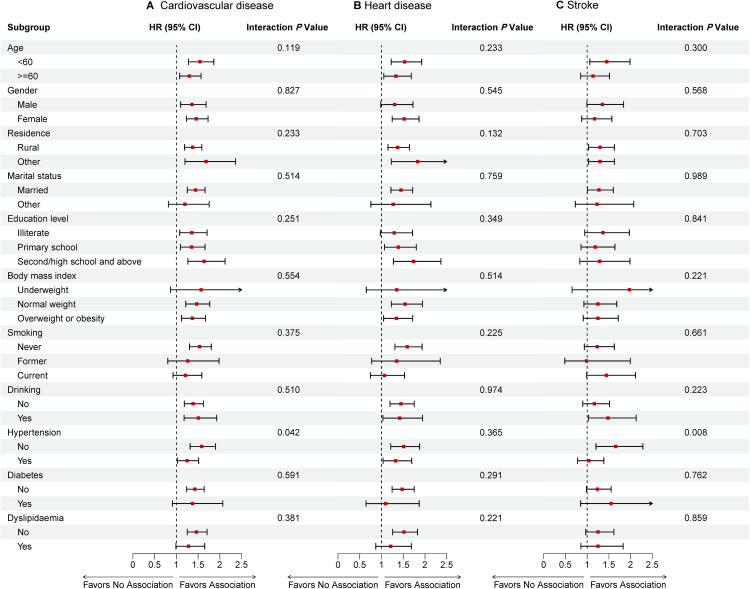
Graphs show hazard ratios (HRs) and 95% CIs for cardiovascular disease (A), heart disease (B), and stroke (C) adjusted for age, sex, residence, marital status, educational level, smoking status, drinking status, body mass index, history of diabetes, hypertension, and dyslipidemia except the stratification factor itself.

**Fig 2** shows the association between loneliness and incident CVD events stratified by potential risk factors. The association between loneliness and incident CVD was more pronounced among participants without hypertension (adjusted HR, 1.582; 95% CI, 1.317–1.900), compared with those with hypertension (adjusted HR, 1.246; 95% CI, 1.027–1.512) at baseline (p = 0.042 for interaction). Similarly, the association between loneliness and incident stroke was more pronounced among participants without hypertension (adjusted HR, 1.657; 95% CI, 1.203–2.281), compared with those with hypertension (adjusted HR, 1.040; 95% CI, 0.781–1.384) at baseline (p = 0.008 for interaction).

When stratifying by gender, we found that the association between loneliness and CVD risk appeared to be slightly stronger in women than in men, although the difference did not reach statistical significance. This may suggest that gender-specific factors, such as hormonal differences or variations in social roles and support systems, could potentially influence the relationship between loneliness and CVD. However, further research with larger sample sizes and more detailed gender-specific measurements is needed to confirm this observation.

### Kaplan-Meier survival analysis

**Fig 3:**
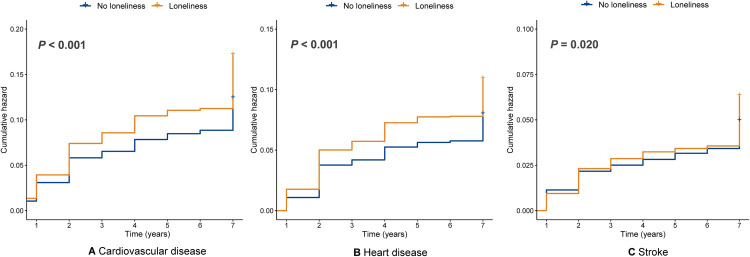
K-M plot of cardiovascular disease (A), heart disease (B), and stroke (C) by loneliness versus no loneliness.

**Fig 3** presents Kaplan-Meier survival curves for CVD, heart disease, and stroke, comparing individuals with loneliness to those without. The curves illustrate the cumulative incidence of each outcome over time, stratified by loneliness status. For all three outcomes, there was a higher cumulative incidence in the group with loneliness compared to those without loneliness, indicating a potentially stronger association between loneliness and the risk of developing CVD (p < 0.001), heart disease (p < 0.001), and stroke (p = 0.02).

### Sensitivity analyses

Sensitivity analyses using inverse probability weighting (IPW) with a fully adjusted model confirmed these findings, with consistent hazard ratios (HRs) across all outcomes. Specifically, the fully adjusted IPW HR for CVD was 1.438 (95% CI, 1.258–1.644), for heart disease was 1.453 (95% CI, 1.232–1.712), and for stroke was 1.283 (95% CI, 1.039–1.585). The findings are detailed in **Table 3**.

**Table 3:** Sensitivity analyses.

Outcome	HR (95% CI)^a^	*p* value	HR (95% CI)^b^	*p* value
Cardiovascular disease				
No loneliness	1 [Reference]		1 [Reference]	
Loneliness	1.377 (1.211, 1.566)	< 0.001	1.438 (1.258, 1.644)	< 0.001
Heart disease				
No loneliness	1 [Reference]		1 [Reference]	
Loneliness	1.367 (1.167, 1.603)	< 0.001	1.453 (1.232, 1.712)	< 0.001
Stroke				
No loneliness	1 [Reference]		1 [Reference]	
Loneliness	1.273 (1.038, 1.561)	0.020	1.283 (1.039, 1.585)	0.021

Abbreviation: HR, hazard ratio.

^a^ Inverse probability of weighting with unbalanced covariates.

^b^ Inverse probability of weighting with age, sex, residence, marital status, educational level, smoking status, drinking status, systolic blood pressure, body mass index, history of diabetes, hypertension, and dyslipidemia.

## Discussion

Our study examined the correlation between loneliness and the occurrence of CVD in a sizable group of middle-aged and older adults in China. After accounting for possible confounding factors, we discovered that loneliness was independently linked to a 42.9% elevation in the risk of CVD occurrence, a 44.7% increase in the risk of heart disease, and a 27.9% rise in the risk of stroke. These results contribute to the expanding collection of evidence connecting loneliness to unfavorable cardiovascular outcomes.

A growing bodyof evidence indicates that loneliness is linked to a heightened risk of CVD. For instance, a cohort study utilizing the Danish National Patient Registry revealed that loneliness is associated with a 1.2-fold elevated risk of cardiovascular morbidity [23]. Another prospective cohort study found that loneliness independently correlates with a moderately increased CVD risk for postmenopausal women, elevating the risk by approximately 1.05-fold [16]. A recent study proposed that for individuals with an obesity index of 1, loneliness is associated with a 1.4-fold greater risk of cardiovascular mortality [12]. Even when accounting for social isolation and loneliness indicators, the obese population showed a lower risk of cardiovascular disease-related mortality [12]. Additionally, multiple meta-analyses and systematic reviews have confirmed a positive association between loneliness and an elevated CVD risk [24–27]. Our study aligns with these findings, revealing a correlation between loneliness and CVD risk, specifically heart disease and stroke. In contrast to some previous research which reported significant gender differences [23,27,28], our study did not find a statistically significant interaction effect of gender on the loneliness-CVD association, although a slightly stronger point estimate was observed in women.

The absence of gender differences in the association between loneliness and CVD risk in our study contrasts with some prior findings. This discrepancy may be attributed to several factors. Firstly, cultural differences in gender roles and emotional expression could play a role. In Chinese society, men and women may experience and cope with loneliness in distinct ways that are not fully captured by the measurement tools used in our study. Secondly, the specific characteristics of our study population, such as the age range and socioeconomic status, might influence the manifestation of gender differences. It is also possible that the relatively small number of CVD events in certain gender subgroups limited our statistical power to detect significant differences. Further research with more diverse samples and nuanced measurement approaches is warranted to clarify this issue.

Although the precise biological mechanisms connecting loneliness to CVD remain incompletely understood, they likely encompass physiological reactions to stress. These reactions could include heightened inflammation, changes in autonomic nervous system functioning, and dysregulation of the hypothalamic-pituitary-adrenal (HPA) axis [29–31]. As a persistent stress factor, loneliness might trigger widespread inflammation and endothelial malfunction—both recognized as risk factors for atherosclerosis and cardiovascular events. More specifically, loneliness could stimulate the HPA axis, leading to raised cortisol levels. These elevated cortisol levels, in turn, could impact blood pressure and endothelial function through the nitric oxide system in blood vessels [32]. This might help explain the link between loneliness and CVD.

Our investigation significantly contributes to the existing literature by revealing a strong correlation between loneliness and the elevated risk of CVD, heart disease, and stroke. This association remains significant even after considering numerous potential confounding factors. Interestingly, this link is particularly pronounced among participants without hypertension, suggesting that blood pressure status plays a crucial role in the relationship between loneliness and CVD risk.

The mechanisms behind this association are likely multifaceted. Firstly, loneliness may affect cardiovascular health through various pathways, including increasing inflammatory responses and altering the function of the autonomic nervous system [11,12]. In the absence of hypertension, these physiological changes may become more apparent as they are not overshadowed by the confounding effects of high blood pressure. Secondly, the psychosocial consequences of loneliness, such as heightened psychological stress and anxiety [33], can indirectly impact cardiovascular health. For those without hypertension, this psychosocial stress may have a more significant effect, as it is not masked by the additional physiological demands of elevated blood pressure. Lastly, loneliness has been linked to unhealthy lifestyle choices, such as reduced physical activity and unhealthy eating habits [34,35], which are known risk factors for CVD. In normotensive individuals, these behavioral factors may have a more substantial impact on cardiovascular well-being.

Our results also revealed that individuals in the loneliness group reported lower rates of alcohol consumption and smoking. This finding may seem counterintuitive at first glance, as one might expect lonely individuals to engage in more health-risk behaviors. However, several explanations could account for this observation. Firstly, lonely individuals may have fewer social opportunities to consume alcohol and tobacco, as these behaviors are often tied to social interactions and gatherings. Secondly, it is possible that individuals who are more health-conscious avoid both social situations involving alcohol and tobacco use and situations that might lead to loneliness. Alternatively, the relationship between loneliness and health behaviors could be bidirectional, with certain health behaviors both influencing and being influenced by one’s social connections. Further research is needed to disentangle the complex interplay between loneliness and health behaviors, and to determine the causal pathways underlying these associations.

Culturally appropriate interventions to address loneliness in Chinese middle-aged and older adults could include community-based social programs that encourage group activities and intergenerational interactions, such as hobby clubs, volunteer opportunities, and educational workshops. These initiatives can provide structured social environments where individuals can build meaningful connections and reduce feelings of loneliness. Additionally, leveraging digital technology to create online social platforms tailored to the needs and preferences of older adults may offer a supplementary approach to enhancing social engagement. Future research should focus on evaluating the effectiveness of such interventions in reducing loneliness and its associated health risks, as well as exploring the long-term sustainability of these programs in different cultural contexts. Furthermore, developing and validating more comprehensive assessment tools for loneliness that account for cultural specifics would strengthen the evidence base for both research and practice in this field.

### Strengths and limitations

The strengths of this study lie in its large, nationally representative sample and prospective longitudinal design. However, there are limitations. One such limitation is the reliance on self-reported measures of CVD, which may be prone to reporting biases and might not fully capture all the clinical intricacies of the conditions. Furthermore, the assessment of loneliness in our study was based on participants’ self-reports, potentially influenced by subjectivity and reporting biases, thereby affecting the precision of loneliness classification and its correlation with CVD. Additionally, there is a possibility of residual confounding stemming from unmeasured or inadequately measured factors like income, the structure of social support, the degree of social isolation, and employment status [36], which could impact the observed associations.

## Conclusion

In conclusion, our findings underscore the notable correlation between loneliness and the elevated risk of incident cardiovascular disease, heart disease, and stroke. Targeting loneliness may prove to be a crucial approach in preventing and managing CVD. However, further studies are required to comprehend the intricacies of this correlation and to develop impactful interventions.

## Supporting information

S1 File Human participant checklist. (DOCX)
